# Early surgery of trochanteric femoral fractures in patients on direct oral anticoagulants (DOACs) reduces the length of stay (LOS) and the 1-year mortality rates without increasing the risk for revision surgery: a matched-pair analysis

**DOI:** 10.1007/s00402-026-06490-2

**Published:** 2026-09-03

**Authors:** Valerie Weihs, Roberta Laggner, Michael Humenberger, Andreas Duma, Martin Frossard, Stefan Hajdu

**Affiliations:** 1 Department of Orthopedics and Trauma Surgery, Medical University of Vienna, Vienna, Austria; 2 Karl Landsteiner University, University Hospital Tulln - NOE LGA, Tulln, Austria; 3 Department of Anesthesia, General Intensive Care Medicine, and Pain Management - Division of General Anesthesia and Intensive Care Medicine, Medical University of Vienna, Vienna, Austria

**Keywords:** DOAC, Hip fracture, Long-term outcome, Mortality

## Abstract

**Background:**

It remains unclear whether early surgical fixation of trochanteric femoral fractures in patients with direct oral anticoagulation (DOAC) therapy has an impact on the long-term outcome.

**Methods:**

A retrospective cohort study on patients with trochanteric femoral fractures and DOAC therapy between 2016 and 2024 was conducted. Patients were divided into two groups according to their time to surgery and matched with age and ASA score as covariates. The primary outcome of interest was the impact of early surgery on the long-term outcome.

**Results:**

Of 233 included patients, 102 patients were assigned in each group after propensity score matching. Patients with early surgery had lower in-hospital complication rates (16.0% vs. 26.3%; *p* = 0.077) and a significantly shorter median length of stay (LOS) (11.5 days (IQR 6) vs. 15 days (IQR 6); *p* < 0.001). No difference in the rates of revision surgery (9.8% vs. 5.9%; *p* = 0.298) was observed. Kaplan-Meier curves revealed a significant difference in the mortality rates during the follow-up period (*p* = 0.0057) with lower in-hospital (2.0% vs. 6.9%; *p* = 0.088), 30-day (3.9% vs. 9.8%; *p* = 0.097) and 1-year mortality rates (20.6% vs. 31.4%; *p* = 0.079).

**Conclusion:**

Early surgical fixation of trochanteric femoral fractures in patients on DOAC therapy leads to a significant reduction in the LOS without increasing the risk for peri- and postoperative complications or the risk for revision surgery. Furthermore, early surgery may positively influence the long-term outcome in this specific cohort of patients.

## Background

 Hip fracture surgery remains one of the most common types of surgery in the elderly and has been shown to increase constantly over the past decades [1, 2]. Not only are the numbers of hip fractures projected to rise further, an increasing number of patients with cardiovascular diseases and active antithrombotic therapy are expected as well [2–4]. As a delay in hip fracture surgery is closely linked to an increase of mortality and complications [5–9], hip fracture surgery is recommended within 24 to 48 h after admission [7, 10–12]. About 20% of patients with hip fractures are known to present with antithrombotic agents on admission [13, 14] with an increase in the use of direct oral anticoagulation (DOAC). Patients with hip fractures and active antithrombotic therapy are at an increased risk for perioperative complications and death. Given this, even in patients with active antithrombotic therapy hip fracture surgery is advised without major delay [15–18] but with the need for precise surgical protocols for hip fracture patients on antithrombotic therapy [19]. So far, little is known about the impact of a DOAC therapy on the outcome of hip fracture patients. A recent study was able to demonstrate higher mortality rates in DOAC patients compared to patients without DOAC therapy irrespective of an early surgery with a one-day delay [20]. To reduce the risk of bleeding events in this cohort of patients, recent international guidelines recommend hip fracture surgery within 24 to 36 h from the last dose of DOAC intake to decrease he percentage of active drugs circulating [21, 22]. In the case of trochanteric hip fractures, a recent prospective study was able to demonstrate that early surgery is feasible without an increase in the perioperative blood loss or transfusion rates [23]. But hip fracture surgery is reported to be possible in only about 40% of DOAC patients within 48 h [24], who showed an increase in mortality associated with the delay of surgery [25]. Our study aims to determine whether the time to surgery in patients on active DOAC therapy and trochanteric fractures affects the length of hospital stay (LOS), the 1-year mortality rate or the need for revision surgery.

## Methods

**Fig. 1:**
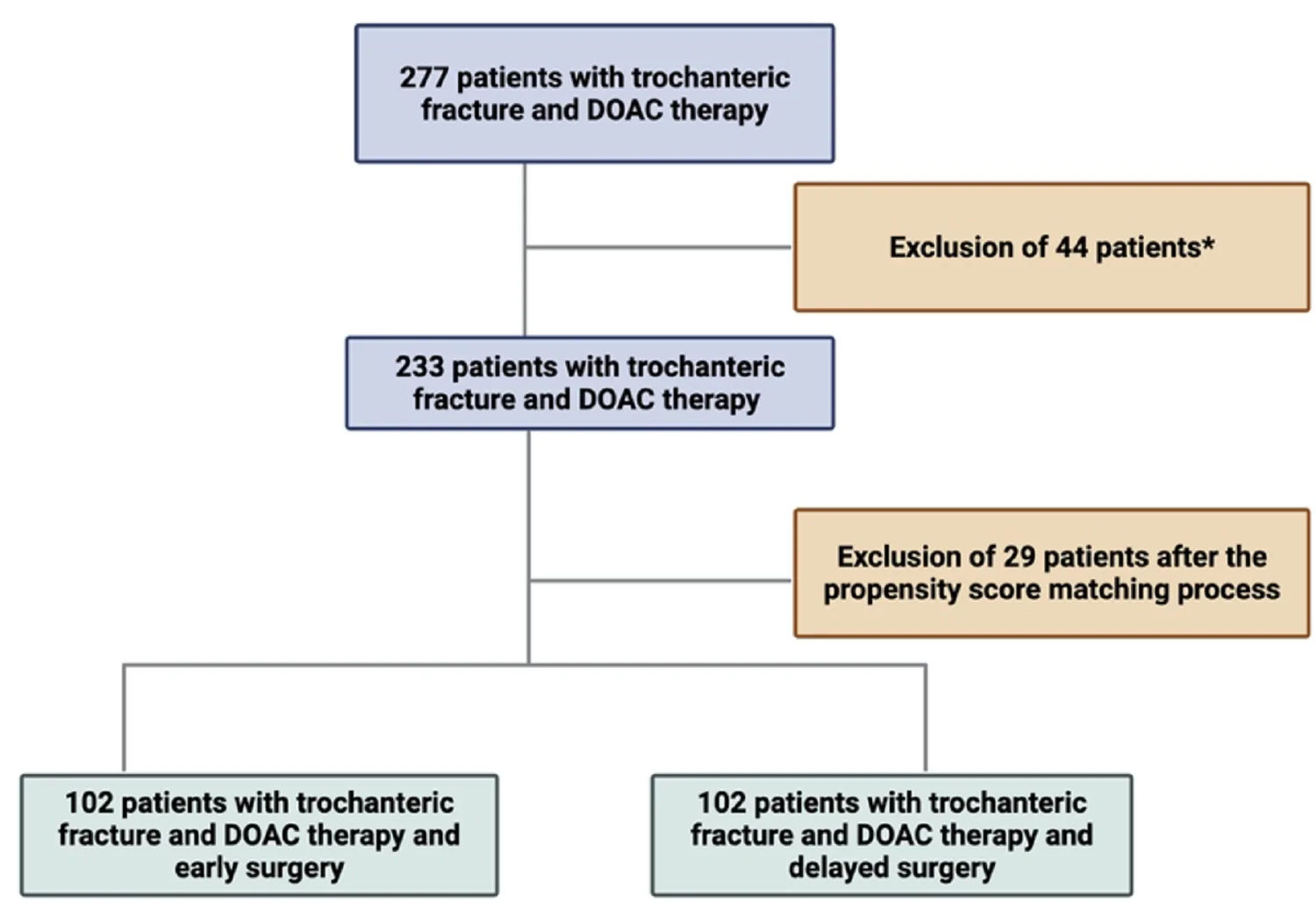
Inclusion of 204 DOAC patients with trochanteric fractures with early surgery (*n* = 102) and delayed surgery (*n* = 102) after 1:1 propensity score matching. *Exclusion of 44 DOAC patients due to: surgical delay for other reasons than DOAC therapy (*n* = 5), missing essential data (*n* = 34), follow up period less than 1 year (*n* = 5). *DOAC* direct oral anticoagulation

Patients with trochanteric fractures under active DOAC therapy were included in this retrospective cohort study. A total of 277 patients were retrospectively included starting from January 2016 to September 2024. Patients were analyzed according to their time to surgery. Early surgery was defined as fracture fixation within 24 h after admission and delayed surgery was defined as fracture fixation later than 24 h after admission. It should be noted that most patients in the early surgery group were included starting from 2022, whereas most patients in the delayed surgery group were included between 2016 and 2021. Early surgical fixation of trochanteric femoral fractures in patients with active DOAC therapy is performed regularly in our department starting at the end of 2021 as part of a prospective observational cohort study that has already been completed. Despite these different time frames, the median follow-up time did not differ between both groups. Exclusion criteria were follow-up less than 1 year (*n* = 5), missing American Society of Anesthesiologist (ASA) score (*n* = 34) and a surgical delay for other reasons than the DOAC therapy (*n* = 5), resulting in a final study cohort of 233 patients (Fig. 1. Created in BioRender. Weihs, V. (2026).

The primary outcome of interest was the influence of time to surgery on the in-hospital, the 30-day, the 90-day and the 1-year mortality rates. Secondary outcome parameters included any kind of in-hospital complication, the length of hospital stay (LOS) and the need for revision surgery (with septic or mechanical failures of the osteosynthesis) during the follow-up period. As baseline demographic details, sex, age, fracture type, ASA score and concomitant comorbidities with the age-adjusted Charlson-Comorbidity Index (CCI) score have been analyzed.

### Statistical analysis

Preliminary statistical descriptive analysis revealed more patients with an ASA score of 4 within the delayed surgery group. To overcome a potential selection bias a propensity score matching with a 1:1 nearest neighboring matching (without replacement) was performed. Covariates that could serve as confounders were defined as age and ASA score. As a result, out of the 233 patients, 204 cases were successfully matched leading to 102 patients in each group (early and delayed surgery) (Fig. 1).

Descriptive analyses were used to evaluate demographic and clinical characteristics. As the continuous variables (age, CCI, time to surgery and LOS) did not meet the criteria for a normal distribution, they are presented as medians and interquartile ranges (IQR). For comparison between categorical variables, Chi-square tests were performed, for the comparison between categorical and continuous variables, Mann-Whitney U tests were done. For the survival analyses different time periods were defined: mortality during the follow-up period, in-hospital mortality (death during the hospital stay), 30-day mortality (death within the first 30 days after the hospital admission), 90-day mortality (death within the first 90 days after hospital admission) and 1-year mortality (death within the first year after hospital admission). An Univariate Cox Regression analysis was performed to identify potential prognostic factors of the 1-year survival. Only variables with a p-value ≤ 0.10 in the Univariate Cox Regression analyses were included in the Multivariate Cox Regression analysis to identify potential independent prognostic factors of the 1-year survival. The statistical analyses were performed using IBM SPSS Statistics Version 29.0. Kaplan-Meier curves were visualized using the statistical software R version 4.5.1 (2025-06-13) [26].

## Results

Most of the patients were female (68.6%; *n* = 140). Intertrochanteric fractures were found in 190 patients (93.1%), whereas subtrochanteric fractures were seen in 14 patients (6.9%). In 87.7% of patients (*n* = 179) an anti-Xa-inhibitor was documented, 25 patients (12.3%) were under therapy with dabigatran. The median age of the patients was 85.5 years (IQR 11; range from 61 to 100 years) with a median CCI of 5 points (IQR 2; range 2 to 11 points). Any kind of complication occurred in 20.1% of patients (*n* = 41) during the hospital stay, whereas revision surgeries were necessary in 16 patients (7.8%). The median LOS was 13 days (IQR 7; range 3 to 98 days). Baseline characteristics of patients with early vs. late surgical fixation are listed in Table 1.

**Table 1:** Baseline characteristics of all matched patients (*n* = 204)

	Early surgery	Delayed surgery	*p* value
	*n* = 102	*n* = 102	
Female n (%)	70 (68.6%)	70 (68.6%)	1°
ASA 2 n (%)	23 (22.5%)	23 (22.5%)	
ASA 3 n (%)	76 (74.5%)	76 (74.5%)	1°
ASA 4 n ( %)	3 (2.9%)	3 (2.9%)	
Age median (IQR)	85.5 (9)	85.5 (10)	0.680*
Intertrochanteric fracture n(%)	98 (96.1%)	92 (90.2%)	
Subtrochanteric fracture n(%)	4 (3.9%)	10 (9,8%)	0.097
Anti Xa inhibitor n (%)	89 (87.3%)	90 (88.2%)	
Dabigatran n (%)	13 (12.7%)	12 (11.8%)	0.831°
CCI median (IQR)	5 (2)	5 (2)	0.715*
TTS median hours (IQR)	12 (11)	46 (25)	< 0.001*
In-hospital complication n (%)	16 (15.7%)	25 (24.5%)	0.077°
LOS median days (IQR)	11.5 (6)	15 (6)	< 0.001*
Revision surgery n (%)	6 (5.9%)	10 (9.8%)	0.298°
Septic n (%)	3 (2.9%)	4 (3.9%)	
Mechanical n (%)	3 (2.9%)	6 (5.9%)	
Follow-up days median (IQR)	696 (659)	708.5 (1172)	0.355*

*CCI* Charlson-Comorbidity Index, *TTS* time to surgery, *IQR* interquartile range; *LOS* length of stay

*Independent Mann-Whitney U test °chi-square test

### Survival analyses

**Fig. 2:**
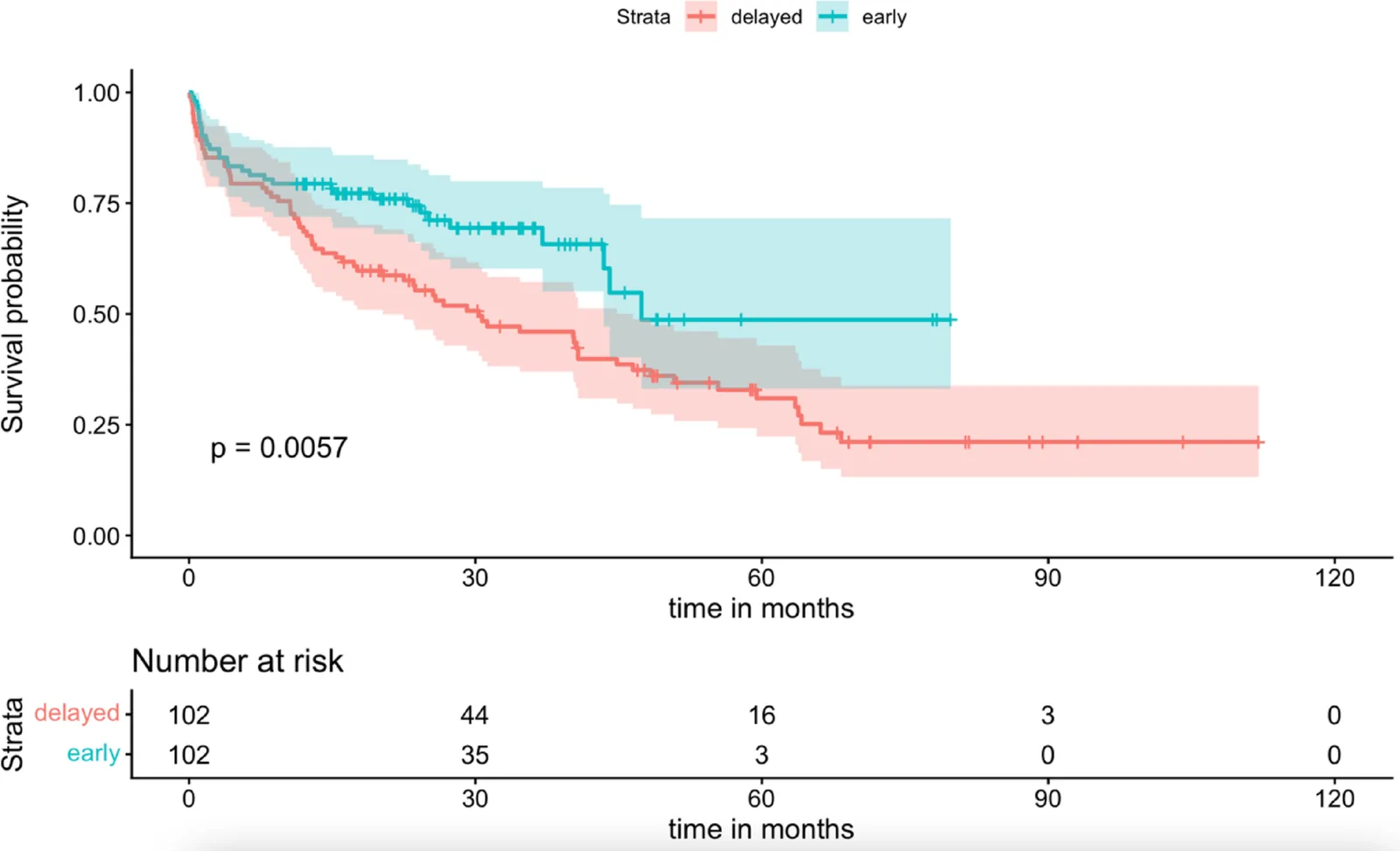
Kaplan–Meier curve with risk table for all matched patients (n=204) and the differences between both groups (early vs. delayed surgery)

A total of 101 patients (49.5%) died during the follow-up period with a median time to death of 348 days (IQR 805, range 2 to 2077 days). In-hospital deaths were documented in 9 patients (4.4%). The 30-day mortality was 6.9% (*n* = 14), the 90-day mortality 13.7% (*n* = 28) and the 1-year mortality was 26.0% (*n* = 53). Patients with early surgical fixation had lower in-hospital mortality rates, lower 30-day mortality rates and lower 1-year mortality rates without reaching statistical significance (Table 2). Kaplan-Meier curves demonstrated significantly lower mortality rates during the follow-up period for patients with early surgical fixation (Fig. 2).

**Table 2:** Differences in the mortality rates in both groups (early vs. delayed surgical fixation)

	Early surgery *n* = 102	Delayed surgery *n* = 102	*p* value
Death at any time *n* (%)	32 (31.4%)	69 (67.6%)	< 0.001°
In-hospital mortality n (%)	2 (2.0%)	7 (6.9%)	0.088°
30-day mortality n (%)	4 (3.9%)	10 (9.8%)	0.097°
90-day mortality n (%)	13 (12.7%)	15 (14.7%)	0.684°
1-year mortality n (%)	21 (20.6%)	32 (31.4%)	0.079°

°chi-square test

The univariate Cox regression analyses revealed potential influences of sex, ASA score and time to surgery on the 1-year mortality. The multivariate Cox regression analyses identified only an ASA score of 4 as an independent prognostic factor for the 1-year mortality (Table 3).

**Table 3:** Univariate and Multivariate Cox regression analyses

	Univariate Cox regression			Multivariate Cox regression		
	HR	95% CI	*p* value	HR	95% CI	*p* value
Sex	0.594	0.344–1.026	0.062	0.608	0.352–1.053	0.076
ASA 3	2.012	0.905–4.474	0.086	1.959	0.880–4.361	0.099
ASA 4	4.228	1.093–16.361	0.037	4.782	1.227–18.630	0.024
TTS ≤ 24 h	0.630	0.363–1.093	0.100	0.616	0.354–1.073	0.087

*ASA* American Society of Anesthesiologists, *TTS* time to surgery, *HR* hazard ratio, *CI* confidence interval

## Discussion

To balance the risk of potential bleeding complications in hip fracture patients under active antithrombotic therapy against the risks of a delay in hip fracture surgery, hip fracture surgery in these patients is recommended within 36 to 48 h [21]. Despite this recommendation, there remains an ongoing debate about the most favourable timing of hip fracture surgery in patients with active antithrombotic therapy [15– [19, 24, 25] and evidence on acute surgery in patients on DOAC therapy is still limited. Early hip fracture surgery appears to be feasible and safe [15– [18, 24] with some studies showing a positive impact of early surgery on patient mortality [27, 28]. Still, most studies on hip fracture patients and antithrombotic therapy are heterogeneous with demonstrate varying results with regards to perioperative complication rates, risk for revision surgeries and 1-year mortality rates. Our study aimed to investigate a homogenous patient population (patients with trochanteric femoral fractures and DOAC therapy) and a minimum follow-up of 1 year.

First, we were able to show a significantly shorter time to surgery (median time to surgery 12 h) and a significant reduction in the LOS (median LOS 11.5 days) in the early surgery group. The delayed group showed a significantly longer time to surgery (median time to surgery 46 h), even though this was consistent with current evidence and local guidelines for the treatment of hip fracture patients under active antithrombotic therapy [7, 10–12].

Second, we observed lower in-hospital complication rates in patient with early surgical fixation. As comorbidities and pre-injury antithrombotic therapy seem to be the most common reasons for delaying hip fracture surgery [29], our results suggest that early surgery for trochanteric hip fractures is feasible and does not only not increase but decrease in-hospital complication rates consistent with recent literature [5–9].

Third, we have observed significantly lower mortality rates in patients with early surgical fixation during the follow-up period. Although the median time of follow-up in both groups was statistically not different, the different follow-up periods of the early and delayed surgery group need to be considered. Additionally, patients with early surgery showed distinctive lower in-hospital, lower 30-day and lower 1-year mortality rates when compared to patients with delayed surgical fixation. Even though these results lack statistical significance, there is a clear trend towards a positive impact of early surgical fixation on the mortality rates of patients with trochanteric fractures and active DOAC therapy.

Fourth, the results of our study demonstrated that early surgery of trochanteric fractures in patients on active DOAC therapy does not lead to an increased number of revision surgeries. During the follow-up period, patients who underwent early surgery had lower revision rates (5.9% vs. 9.8%), but this difference does not reach statistical significance. Given the assumption that patients with active DOAC therapy are under an increased risk for fracture-related haematoma formation, early surgery of an unstable trochanteric fracture might serve as a preventative factor for perioperative bleeding complications and subsequently the need for revision surgeries.

We are facing a constant increase in the burden of hip fractures globally [1– [3, 30] together with an increasing proportion of patients with cardiovascular diseases and active antithrombotic therapy [2–4]. This correlation presents a growing challenge for orthopaedic surgeons nowadays, as we are now facing not only an increasing number of hip fractures but also an increasing proportion of frail patients with multiple comorbidities.

The results of our study suggest that early surgery of trochanteric fractures in patients with DOAC therapy reduces the LOS significantly without increasing the risk for revision surgeries or mortality rates.

### Limitations of this study

Despite careful analysis, this study has some limitations. Although we aimed to present a homogeneous study cohort, there might be a selection bias due to the retrospective design of this study. The patients’ records have been reviewed carefully to determine the causes of a surgical delay and only patients who had a surgical delay due to the anticoagulation therapy have been included. Patients with any other reason for a surgical delay have been excluded from this study. Therefore, we might have missed a potential benefit of a surgical delay in patients in whom a medical optimization has been possible. Furthermore, another limitation of this study is that patients were included during the Covid-19 pandemic, which might be a potential bias. We were able to present a long-term follow-up of at least 1 year in all included patients, but the study might be underpowered to demonstrate the potential benefit of early surgery on the mortality of these patients. Future research with prospective, potentially multi-center trials is needed to find the optimal timing of surgery for hip fractures in general in the individual patient with antithrombotic therapy.

## Conclusion

This retrospective cohort study showed that early surgical fixation of trochanteric fractures in patients on DOAC therapy significantly reduces the length of hospital stay without increasing the postoperative complication rates. According to our results, early surgical fixation is recommended in patients on DOAC therapy as it does not increase the risk for revision surgery and the time to surgery might be a crucial factor for an improved outcome in these patients.

## Data Availability

The datasets generated and analyzed in the current manuscript are not publicly available due to data protection regulations. The data used and analyzed in this study are available from the corresponding author solely upon reasonable request.
